# Inkjet Printing Humidity Sensing Pattern Based on Self-Organizing Polystyrene Spheres

**DOI:** 10.3390/nano10081538

**Published:** 2020-08-05

**Authors:** Valeriia O. Neterebskaia, Anna O. Goncharenko, Sofia M. Morozova, Denis S. Kolchanov, Alexandr V. Vinogradov

**Affiliations:** Inkjet printing group, International Institute “Solution Chemistry of Advanced Materials and Technologies” (SCAMT), ITMO University, 191002 Saint Petersburg, Russia; vneterebskaya@scamt-itmo.ru (V.O.N.); Goncharenko@scamt-itmo.ru (A.O.G.); morozova@scamt-itmo.ru (S.M.M.); kolchanov@scamt-itmo.ru (D.S.K.)

**Keywords:** photonic coatings, photonic crystal, polystyrene spheres, chitosan hydrogel, inkjet printing

## Abstract

This study is devoted to the development of photonic patterns based on polystyrene spheres (PSS) incorporated in chitosan hydrogels by inkjet printing. Using this method, high-resolution encrypted images that became visible only in high humidity were obtained. Inks based on PSS with carboxylic groups on the surface were made, and their rheological parameters (viscosity, surface tension, and ζ-potential) were optimized according to the Ohnesorge theory. The obtained value of the ζ-potential indicated the stability of the synthesized colloidal inks. The dependences of the printing parameters on the concentration of ethylene glycol in PSS dispersion, the drop spacing, the shape of the printed pattern, waveform, the temperature of the printing process, and the degree of ordering of the PSS-based photonic crystal were investigated. The scanning electronic microscope (SEM) images confirmed that the optimal self-organization of PSS was achieved at the following values of 0.4% weight fraction (wt%) carboxylic groups, the drop spacing of 50 μm, and the temperature of the printing table of 25 °C. High-resolution microstructures were obtained by drop-on-demand printing with a deposited drophead diameter of 21 μm and an accuracy of ±2 μm on silicon and glass substrates. The deposition of chitosan-based hydrogels on the obtained polystyrene photonic crystals allowed reversibly changing the order of the diffraction lattice of the photonic crystal during the swelling of the hydrogel matrix, which led to a quick optical response in the daylight. The kinetics of the appearance of the optical response of the obtained coating were discussed. The simplicity of production, the speed of image appearance, and the ability to create high-resolution patterns determine the potential applications of the proposed systems as humidity sensors or anticounterfeiting coatings.

## 1. Introduction

Photonic crystals are materials with an ordered structure characterized by strict periodic changes in a refractive index at scales comparable to wavelengths of radiation in the visible and near-infrared ranges [1,2,3]. Nowadays, the most studied and advanced photonic crystals are opal-based ones, which can be regarded as “traditional” [3]. Such materials are of great interest owing to their wide applications in optics [4,5], different sensors [6,7,8,9], in vivo imaging [10], and anticounterfeiting labels [11]. Among these applications, the formation of structural colors for anticounterfeiting and information storage has growing importance.

Polystyrene spheres (PSS) are prospective and well-studied materials for the formation of photonic crystals due to their cheapness, simplicity of synthesis, easy controlling of their size and distribution of particles by size, colloidal stability, and high refractive indices [12]. The coating of photonic crystals with hydrogels allows obtaining stimuli-responsive materials that can change the diffraction lattice of the photonic crystals under the variation of pH [13], humidity [14], ionic strength [15,16,17], or other external impacts.

There are different ways to form photonic crystals based on PSS. Du et al. using drop-casting for the preparation of photonic crystals images [14]. Covering them by chitosan (CS) hydrogels leads to the formation of coatings, and the dependence of the optical response from red to green colors on the size of PSS has been demonstrated. Other techniques include the control of drying conditions—colloidal crystalline poly(styrene-co-acrylic acid (AA)) microspheres, which are formed as water in emulsified droplets that evaporate leaving behind closely packed lattices of polyelectrolyte core–shell particles [15,16]. Photonic crystals based on the mentioned microspheres are responsive to the ionic strengths of the media of cationic electrolytes and have been applied for in vivo imaging of the morphology and the concentration gradient of cationic electrolytes along the gastrointestinal tract of live Japanese medaka [17]. In addition, a method for obtaining self-organized PSS by dip-drawing was also presented [13,16,17]. The Obtained photonic crystals were highly ordered, and the dependence of structure color on the size of used spheres was demonstrated. Zhang et al. fabricated the arrays of polystyrene (PS) colloidal particles at the air/water interface by the needle tip flow method [13]. The obtained photonic crystals showed pH-responsible optic response varying from green (pH = 7) to red (pH = 5) colors. The integration of a response stimulus is an additional advantage for the application of photonic crystals in combatting counterfeiting. However, it is also necessary to consider the need to create unique high-resolution images based on such coatings, which is difficult to achieve by the methods listed above.

In this regard, inkjet printing is a promising method for fast manufacturing large-scale high-resolution photonic coatings in an inexpensive way [18,19,20,21]. It is a noncontact deposition technology and avoids impurities or substrate breakdowns. According to the drop-on-demand technology, it allows creating high-resolution templates upon several micrometers [22]. Despite the fact that the droplet size is limited to the micrometer scale, it is possible to achieve self-organization of nanoparticles into a photonic crystal due to the control of printing parameters, ink composition, and drying conditions [23,24,25]. A modification of this method is dual-drop printing, in which a supporting drop is printed followed by nanoparticles-based inks [26]. Despite the fact that the self-organization of PSS occurs at the interface between the surfaces of two drops and allows obtaining nearly monolayer photonic crystals without a coffee ring effect, this method is rarely used because it requires more complex parameters of printing and special equipment than the conventional inkjet technique [26,27].

Previously, our group developed ink for inkjet printing of PSS [19]. However, for the use of PSS in stimuli-responsive coatings, it is necessary to introduce functional groups, usually AA, into them for better compatibility of hydrophobic PS with hydrophilic hydrogels. Because printing parameters, such as drop spacing (DS), substrate temperature, drop speed. are unique for each new ink, the ways to control the self-organization conditions of PSS during the drying of droplets should be studied for functionalized PSS [27,28].

Thus, in this work, photonic humidity-sensitive coatings based on PSS incorporated in CS hydrogels were obtained by inkjet printing for the first time. We have investigated the influence of inkjet-printing parameters on the self-organization of PSS. The printed complex patterns provide fast and reversible color response due to the humidity changing and could be used as humidity sensors or anticounterfeit coatings.

## 2. Materials and Methods

### 2.1. Reagents

Styrene (St, 99.8%, Sigma-Aldrich, Schnelldorf, Germany), potassium peroxodisulfate (KPS, K_2_S_2_O_8_, 99.8%, Sigma-Aldrich, Schnelldorf, Germany), AA (99.8%, Sigma-Aldrich, Schnelldorf, Germany), CS (99.8%, Sigma-Aldrich, Schnelldorf, Germany), ethylene glycol (EG, 99.5%, Vekton, St. Petersburg, Russia), glutaraldehyde (50%, Sigma-Aldrich, Schnelldorf, Germany), acetic acid (99.5%, Vekton, St. Petersburg, Russia), and sodium hydroxide (98%, LenReaktiv, St. Petersburg, Russia) were used as received without further purification. Deionized water was used in all the experiments.

### 2.2. Characterization

Particle sizes and the zeta potential of PS dispersion and inks were measured by dynamic light scattering (DLS) on Photocor Compact-Z (Moscow, Russia). The scattering angle was 90°, and the laser power was 25 mW. Rheological measurements were performed on a rotational viscometer (Fungilab Expert, Barcelona, Spain) with a small sample adapter APM. Surface tension and contact angle were measured on a drop shape analyzer (KRUSS DSA-25, Hamburg, Germany). Morphology images were obtained using a scanning electronic microscope (SEM; Tescan Vega 3, Brno, Czech Republic) and an atomic force microscope (AFM NT-MDT NEXT, Moscow, Russia). Transmittance spectra were obtained using a spectrophotometer (Cary 8454 UV-Vis, Santa Clara, CA, USA). The Fourier transform was performed using the ImageJ software (win64, National Institutes of Health, USA).

### 2.3. Preparation of Inks and Printing

Printable inks for inkjet printing were prepared from 11.2 wt% PS colloids and EG. The FujifilmDMP-2831 printer with a waveform editor and a drop-watch camera was used in this work. Its system allowed for the manipulation of electronic pulses to a piezoelectric jetting device for the optimization of the drop characteristics, as it was ejected from the nozzle. Cartridge models #DMC-11610/PN2100201146 with a capacity of 1.5 mL were used for printing. Cartridges can easily be replaced to support the printing of a series of fluids.

### 2.4. Preparation of Substrates

Silicon wafers (size: 3 inch × 0.387 mm; roughness: 1 nm) (Sigma-Aldrich) and glass slides (size: 76 mm × 26 mm × 1 mm; roughness: <0.15 μm) (MiniMed) were cleaned sequentially with detergent, pure water, acetone, and isopropanol in an ultrasound bath for 15 min. Then, the slides were dried in a flow of nitrogen.

### 2.5. Preparation of Hydrogel Coating

CS was dissolved in 2 wt% acetic acid. Then, it was neutralized with a 0.4 M NaOH solution. To create a thin CS film, methods such as drop-by-drop casting, dip-coating, and spin-coating were considered. After a thin film of the CS solution (3.0 wt%) on the surface of the sample was deposited, the sample was placed for 2 h under UV light and then in an oven at 50 °C for 2 h to let water evaporate. Finally, a small amount of a solution with low-concentration glutaraldehyde (0.25%) was added rapidly (for 5 s) to the sample, which was printed on glass, followed by washing with water.

### 2.6. Study of Optical Properties of Photonic Coating

A sample printed on optical glass (the choice was related to the substrate transparency) was fixed in a spectrophotometer, and the values of all wavelengths were taken in equal time ranges with an interval of 10 s.

## 3. Results and Discussion

### 3.1. Ink Formulation

**Figure 1:**
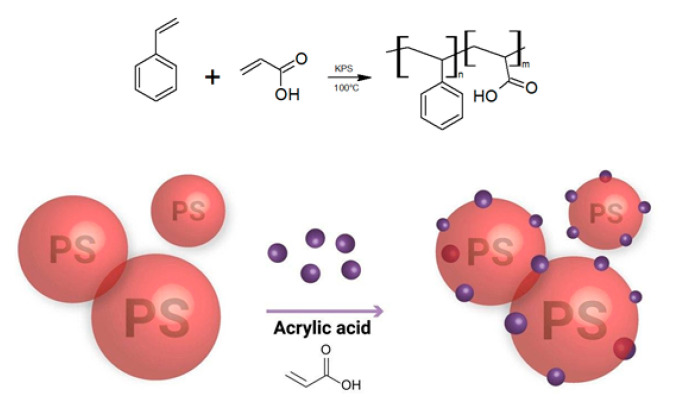
Schematic illustration of the synthesis process for the preparation of a polystyrene (PS)– acrylic acid (AA) copolymer.

The synthesis of a copolymer of PS with AA (0.2, 0.4, and 0.6 wt%) illustrated in Figure 1 was performed following the method reported earlier by Du et al. [14].

The size of the PSS according to the DLS and SEM data was 100 nm in diameter with a size distribution of 83.5% (Figure S1). The choice of the size of the spheres was explained, on the one hand, by the need to avoid clogging the print head in the case of particle aggregation, which required the size as small as possible; on the other hand, self-organization into the photonic crystal of the sphere was better in the case of a size of several hundred nanometers [6]. The presence of carboxyl groups from AA, which are necessary for better binding to CS hydrogels, was confirmed by the presence of the corresponding characteristic bands on the IR spectrum (3000–3030 cm^−1^ νOH, 1705 cm^−1^ νC=O; Figure S2). The dependence of the self-organization of PSS on the drying temperature (Figures S3 and S4), the concentration of AA (Figure S5), and the application method were studied (Figure S6). Drop-casting patterns were also obtained to confirm the concept (Figure S7). According to the data obtained, the best results were shown by spheres with an AA content of 0.4% when drying at 25 °C.

**Figure 2:**
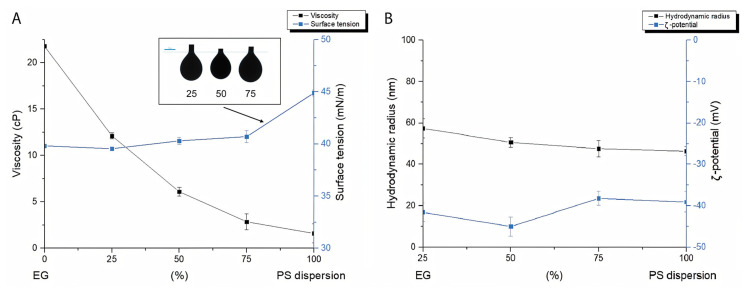
Rheological parameters for inks with different ratios of polystyrene spheres (PSS)/ethylene glycol (EG): (**A**) dual system diagram (PS, EG) describing the relationship between the percentages of components and parameters such as viscosity and surface tension; (**B**) dual system diagram (PS, EG) describing the relationship between the percentages of components and parameters such as hydrodynamic radius and ζ-potential.

In the inkjet printing technology for droplet formation, the liquid must have specific rheological properties including given values of the Ohnesorge theory, which is related with Reynolds and Weber numbers [29]. The synthesized PSS suspension had a low viscosity that did not allow it to reach the printing window with the Ohnesorge number in the range from 0.1 to 1. To determine the optimal rheology (viscosity, surface tension, hydrodynamic radius, and zeta potential), various concentration ratios of PS to EG were investigated (Figure 2).

As can be seen from Figure 2, it was possible to achieve significant changes in the value of the viscosity parameter, which increased noticeably from 1.6 to 21.8 cP with the addition of EG. The hydrodynamic radius of the particles increased slightly with the increasing of EG concentration. In addition, there was an insignificant decrease in the value of surface tension, while the stability values reflected by the zeta potential of PSS suspension remained almost unchanged. From these curves, the optimal PSS/EG ratio of the ink’s components close to 50/50 was chosen. The optimized ink was characterized with the following parameters: a viscosity of 5.6 cP, a surface tension of around 40.5 mN m^−1^, a hydrodynamic radius of 50 nm, and a zeta potential of about 42 mV.

### 3.2. Inkjet Printing Parameters

**Figure 3:**
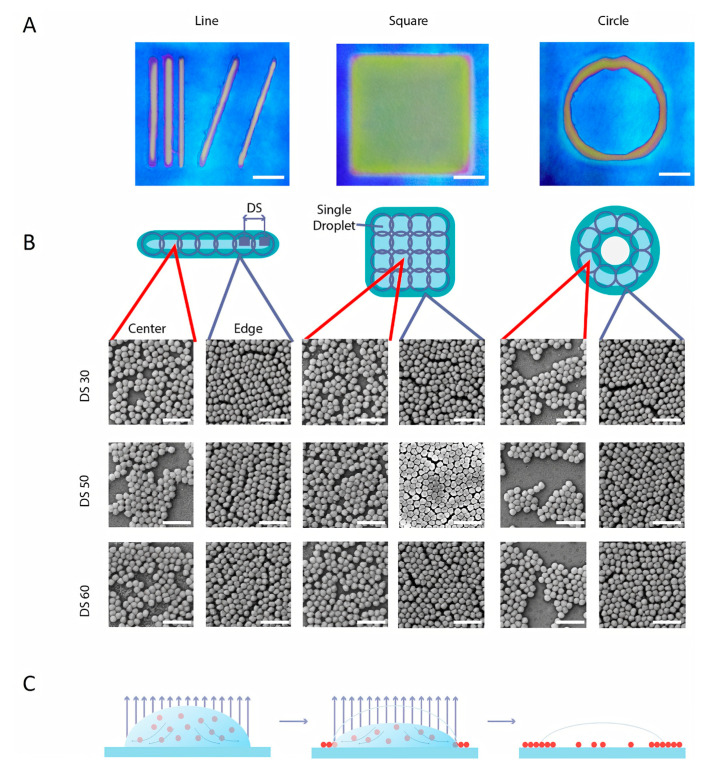
(**A**) The images of 3 types of printed patterns. Scale bar: 1 mm. (**B**) SEM images of PS ordering in the center and on the edge of the droplet in the printed forms with different drop spacings (DSs). Scale bar: 500 nm. (**C**) The distribution of matter in a drop by taking into account the Marangoni effect.

In order to optimize conditions for obtaining PSS-based photonic crystals, it was necessary to maintain a balance between two opposite points: the more ordered the photonic crystal, the brighter optical response it will be able to give, but the more ordered the crystal, the more complex, expensive and time-consuming the technology to produce it. The inkjet printing technique is a simple, fast and efficient way to create complex patterns based on ordered nanoparticles. However, the printing settings play a significant role in the processes of their self-organization. The effect of DS on the degree of the self-organization of the PSS was investigated for three types of printed patterns (line, square, and circle), and the SEM images of the respective photonic crystals were analyzed (Figure 3a).

**Figure 4:**
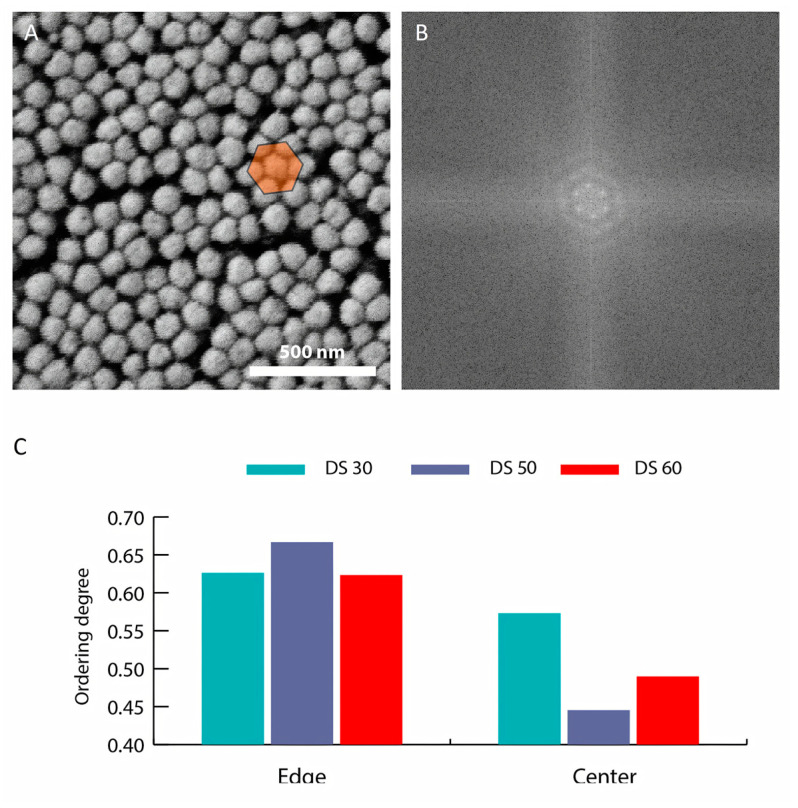
(**A**) The SEM image of the sample. (**B**) The Fourier transform of the ordered PSS. (**C**) The histogram of the distribution of the degree of ordering at the edges of and in the center of the printed structures, depending on the DS.

Then, a line of samples was created by changing the distance between the centers of the droplets from 25 to 75 μm. On the one hand, if the DS was less than the drop diameter, then PSS had the less contact with each other and the uniform gradual drying led to the increasing of the level of PSS ordering (Figure 3b). On the other hand, it is important to get a clear image of the desired pattern. If the DS was too short, the printed pattern became blurry, while a very large DS made the image torn (Figure 3b). It is also worth noting that the degree of self-organization differed on the edge of and in the center of the drop due to the Marangoni effect (Figure 3c). The degree of ordering of PSS into a photonic crystal with the close-packing of equal spheres, depending on the drying temperature, was estimated by the Fourier method (Figure 4B) and by the density of the particle distribution (Figure 4C).

Quantitatively, the level of self-organization was estimated by studying the density of space-filling with particles. Considering that the densest packing of spherical particles fills the space of a plane by 90.69% [30], we accepted that this density is characteristic of a perfectly self-organized structure. Using the ImageJ software package, we converted the drawings to a 1-bit format (Figure S8) and found out the density of space-filling with PSS. The self-organization coefficient was obtained by dividing the data obtained and the values of the densest packages. The greatest order was observed at the edges of the printed structures, which were characterized by a hexagonal packing of particles and confirmed by the Fourier analysis (Figure 4A,B). For the printed samples, a DS of 50 μm was selected, since these samples showed the maximum ordering at the edges of the structures and low particle density in the center of the structures (Figure 4C).

### 3.3. Hydrogel Formation on the Substrate

**Figure 5:**
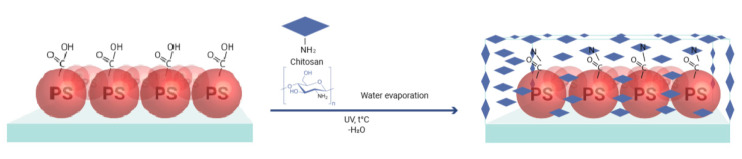
Schematic illustration of the crosslinking process of the PS–AA copolymer with chitosan.

To obtain a stimuli-responsive photonic coating, a layer of a CS hydrogel was deposited on the top of the printed PSS pattern (Figure 5).

Several ways to create a thin CS film were considered: (i) drop-casting, (ii) dip-coating, and (iii) spin-coating. Unlike the first two methods, the spin-coating yielded the most uniform and thin film of CS. However, images obtained by drop-casting and dip-coating were brighter than spin-coating analogues. This may be due to the disordering of the structure of the photonic crystal under the action of a centrifugal force during spin-coating. The depositions by the drop-casting method as well as the dip-coating gave approximately the same optical effect. As a result, the dip-coating technology was chosen, because of its scalability, faster deposition, the absence of the probability of mechanical damage of the image (scratching the sample with a syringe needle), and the increased uniformity of the applied layer. It was found that the ratio of PSS to the hydrogel affected the optical signal. There was a minimum PSS:hydrogel ratio of 0.5:1, at which a signal can be seen (Table S2), and there was a maximum PSS:hydrogel ratio (1:6), probably limited by the diffusion of water through the hydrogel to the photonic crystal (Table S2).

**Figure 6:**
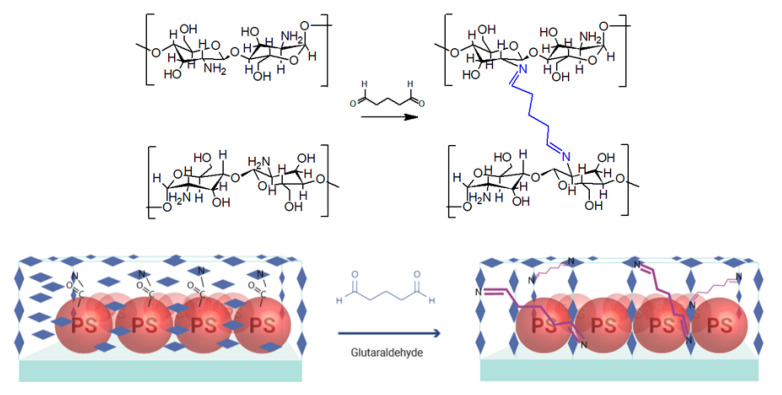
Scheme of the crosslinking of chitosan with glutaraldehyde.

To prevent the dissolution of the hydrogel and create a reversible response, the crosslinking of CS with glutaraldehyde was carried out. A small amount of the solution with 0.25 wt %-concentration glutaraldehyde was added rapidly (for 5 s) to the sample, which was printed on glass, followed by washing with water. The bond between CS and glutaraldehyde increased the pattern reusability and thermal stability. Figure 6 shows the process of crosslinking.

In the case of printing on a silicon substrate, crosslinking was not necessary, because binding occurred due to the COOH groups of PSS, the NH_2_ groups of CS, and the OH groups of the silicon wafer. This could be explained by different PS/CS ratios on the glass and on the silicon substrate, which plays an important role in optic response (Tables S1 and S2). CS attached less with hydrophobic silicon, and its cross-linking with glutaraldehyde led to a significantly tightly crosslinked system. In this case, after adding water, the image was not observed at all. While on the glass, CS was deposited better, and its crosslinking with the same amount of glutaraldehyde was less (the ratio of glutaraldehyde/CS was smaller than on a silicon substrate).

### 3.4. Optical Humidity Response

**Scheme 1:**
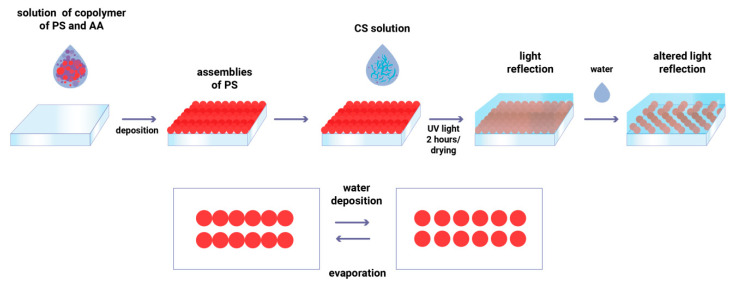
Schematic illustration of the photonic coating creation. An image was displayed and hidden before and after water interaction, respectively.

The production of a photonic coating with a full optical humidity response is shown in Scheme 1.

**Figure 7:**
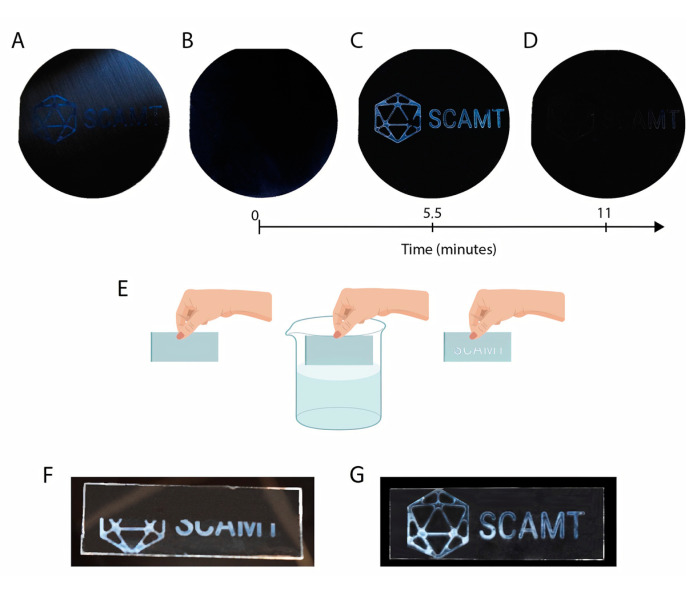
The photos of printed PS colloid on a silicon wafer (**A**), the PS–CS hydrogel before humidity changes (**B**) image appearance after water addition (**C**), and hydrogel after complete drying (**D**). (**E**) Schematic illustration of the process of image appearance after water interaction. The photos of a hydrogel sample on a glass slide after half of the sample was submerged in water (**F**) and after the whole sample was submerged in water (**G**).

A pattern based on PSS ink was deposited on the corresponding substrate, which formed a photonic crystal when it was dried. Then, the image was coated with CS and crosslinked with glutaraldehyde as necessary, and a hidden image was obtained in the daylight. When wet, the hydrogel swelled and increased the distance between the PSS, as they were connected to the hydrogel to form an optic response. We implemented this scheme for the printed logo of the scientific laboratory SCAMT on glass and silicon substrates (Figure 7).

To test the formation of a photonic coating response after interaction with water, the following methods were studied: water spraying, drop-by-drop application, and the immersion method. The brightest optical response was achieved with the last method, which included rapidly dipping the sample in a water tank. The influence of the substrate was also studied: in both cases (silicon and glass substrates), a strong optical response was observed after wetting the samples. The possibility of obtaining optical response was observed during five cycles of water deposition and drying, which may be due to a violation of the order of the structure of the photonic crystal. For each subsequent cycle, the image was saved for the first 2–3 times with slight changes. For the 4th time partial, local deformation was observed, and on the 5th cycle, the pattern became unreadable. The weak dependence of the optical response on the amount of water (spray technique) made the proposed photonic coating show low sensing performance. However, the bright optical response with the submersible technology and its renewability to five cycles makes photonic coatings prospective as anticounterfeit products.

**Figure 8:**
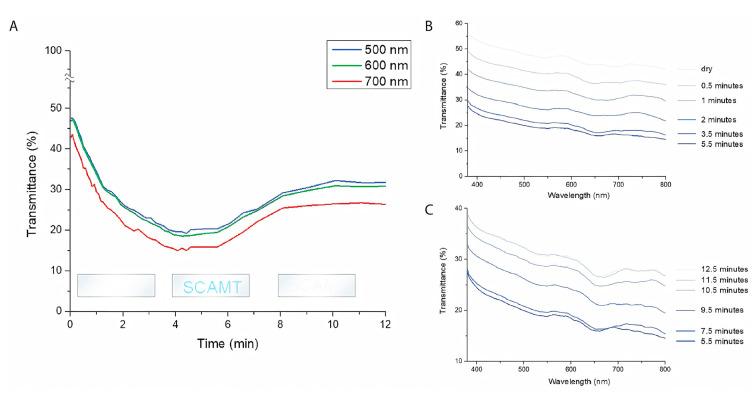
The graphs of the kinetic dependences of the transmittance on time at the wavelengths of 500, 600, and 700 nm (**A**), wavelength after adding water (**B**), and the gradual drying of the sample (**C**).

After coating the sample with CS, kinetics experiments were performed. The appearance of optical response was confirmed by transmission spectrometry (Figure 8).

In Figure 8A, the kinetics of the light transmission of the sample after adding water were demonstrated. The tendency of the light transmission deterioration was clearly traced by observing the appearance of a latent image. After 5.5 min, the image began to disappear again, which was also confirmed by the increase in the transmission line with time (Figure 8B). The visible response was noticed after 30 s. After the initial humidity level was restored, the image completely disappeared for the next 12 min.

## 4. Conclusions

Inks based on PSS with carboxylic groups on a surface were obtained, and their rheological parameters were optimized for their suitability for inkjet printing by adding EG to obtain a PSS:EG ratio of 10:9. It was shown that the degree of ordering of PSS into photonic crystals could be controlled by the variation of the printed pattern, DS, and the temperature regime of drop-drying. The optimal printing parameters for high-quality image processing and high-degree self-assembly of PSS were shown as following: a DS of 50 μm and a temperature of 25 °C during drying. After the comparison of deposition techniques for CS on PSS-based printing patterns, the most uniformity of the film and high optic response after hydrogel swelling were achieved by dip-coating. High-resolution patterns were formed using inkjet printing, which were able to give a reversible optical response under changes in humidity, and observed 30 s after applying the stimulus. Due to the rapid process of creating photonic coatings by the inkjet printing technology, the use of water as a nontoxic image developer, and the complexity of the reproducibility of the technology, these hydrogels can be promising materials for anti-counterfeiting manufacture and humidity sensors.

## Supplementary Materials

The following are available online at https://www.mdpi.com/2079-4991/10/8/1538/s1, Figure S1. The size distribution of the synthesized PSS measured by the dynamic light scattering method, Figure S2: The IR spectrum of a copolymer of PS and AA, Figure S3: SEM images of the PS solution with 0.2 wt% of AA in 3 types of drying regime, Figure S4: SEM images of the PS solution with 0.4 wt% of AA and SEM images of PSS taken from the surface of water, Figure S5: SEM images of PS with different AA contents, Figure S6: SEM images and the scheme of the deposition of the PS solution with a 0.2 wt% AA content. Figure S7: Photographs of a hydrogel before and after humidity changes. Figure S8: Images describing the dependences of the self-organization of PSS on the shape of the printed pattern and the location of the spheres, Table S1: Different ratios of chitosan and glutaraldehyde for the optimal hydrogel formation, Table S2: The influence of the hydrogel composition on the sensitivity to humidity changes.
