# Supplementary material for: Inkjet Printing Humidity Sensing Pattern Based on Self-Organizing Polystyrene Spheres
Source: Nanomaterials (Basel). 2020 Aug 5;10(8):1538. doi: 10.3390/nano10081538 (PMC7466399; doi:10.3390/nano10081538)
Supplement: Supplementary file 1 [file nanomaterials-10-01538-s001.pdf]

# Supporting Information

## Inkjet Printing Humidity Sensing Pattern Based on Self-Organizing Polystyrene Spheres

Valeriia O. Neterebskaia, Anna O. Goncharenko, Sofia M. Morozova, Denis S. Kolchanov and Alexandr V. Vinogradov

Inkjet printing group, International Institute "Solution Chemistry of Advanced Materials and Technologies" (SCAMT), ITMO University, Saint Petersburg, 191002, Russian Federation

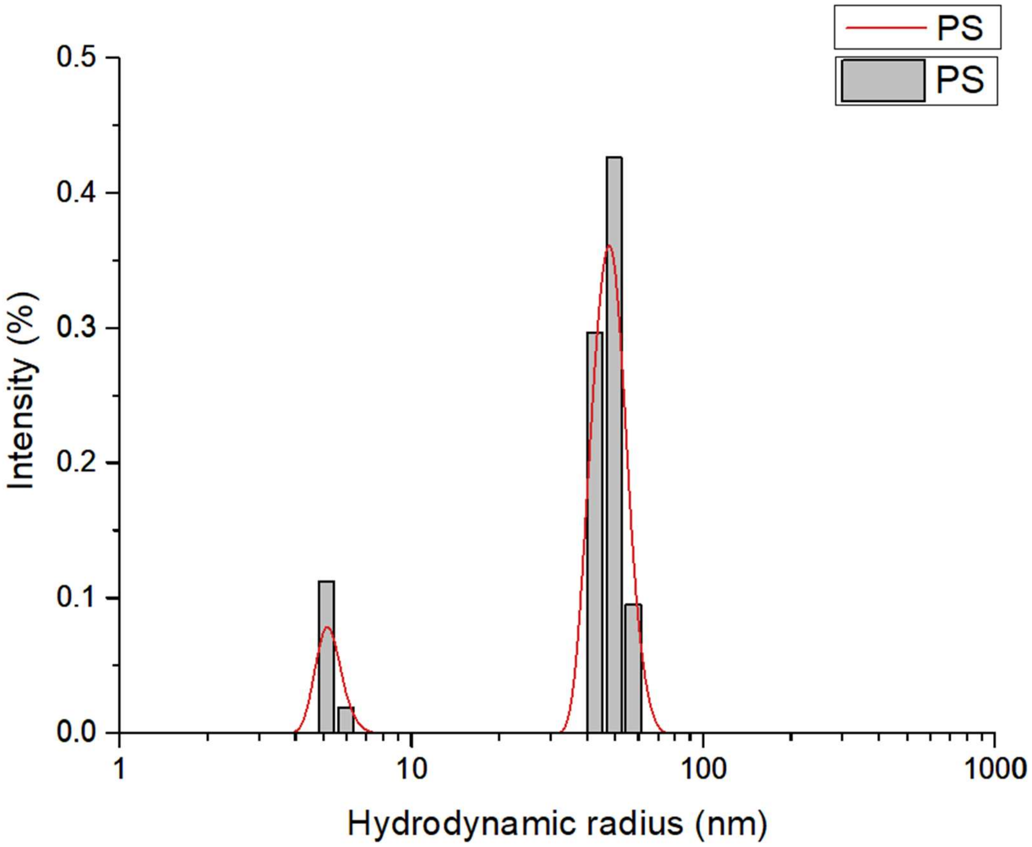


**Figure S1.** Size distribution of the synthesized polystyrene spheres measured by dynamic light scattering method.

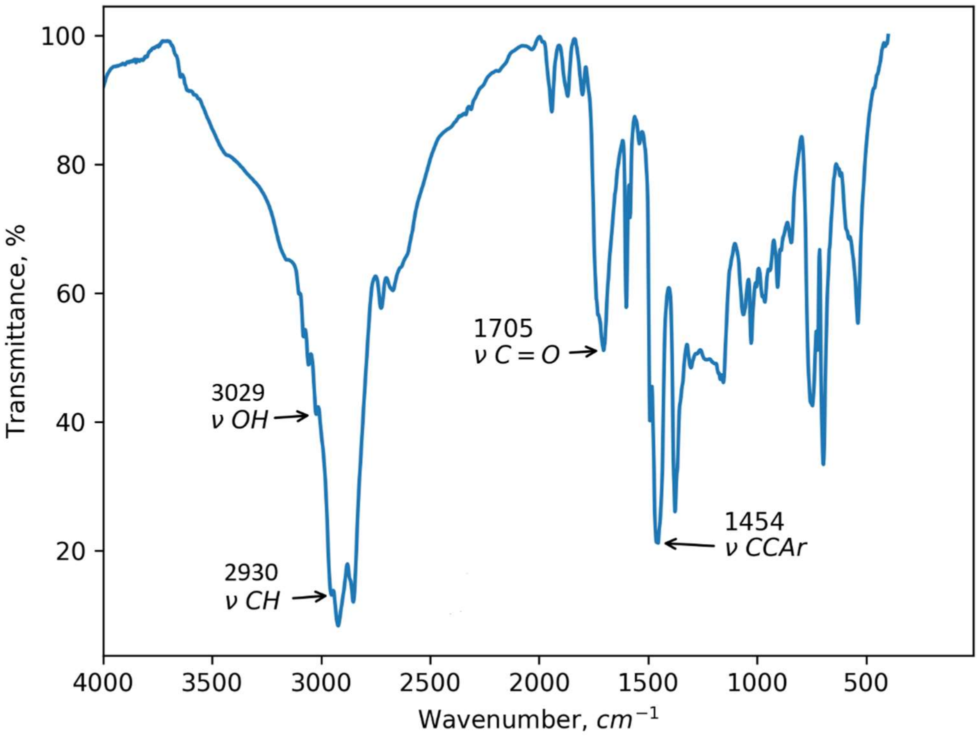


**Figure S2.** IR spectrum of a copolymer of polystyrene and acrylic acid.

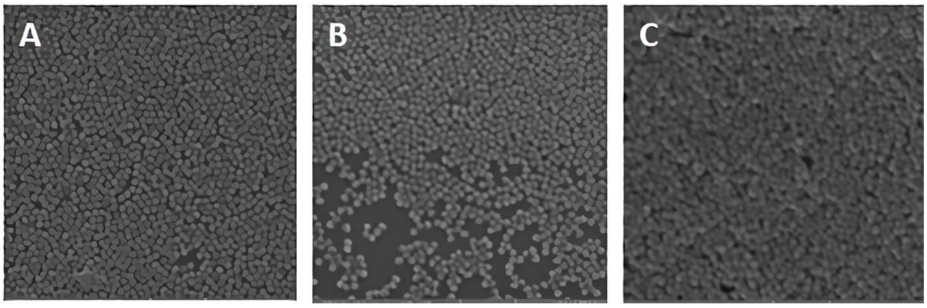


**Figure S3.** SEM images of PS solution with 0.2 wt% of AA in 3 types of drying regime. A) drying at 25°C B) drying at 50°C C) drying 25°C at vacuum.

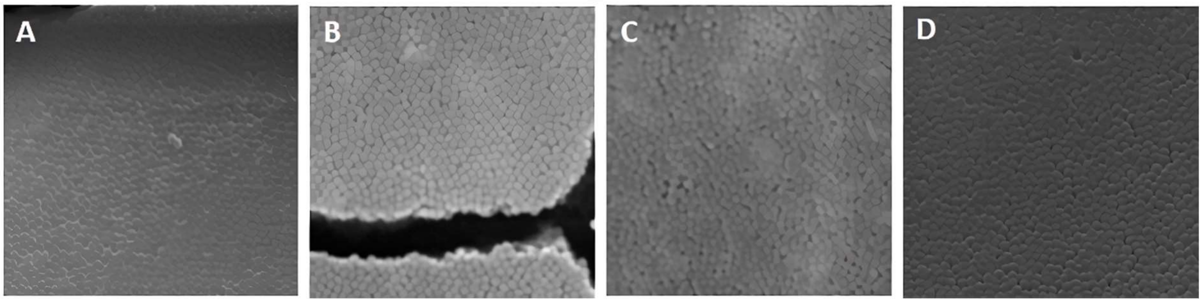


**Figure S4.** SEM images of PS solution with 0.4 wt% of AA SEM-images of PS spheres taken from the surface of water whose temperature is A) 25°C B) 35°C C) 50°C D) 65°C.

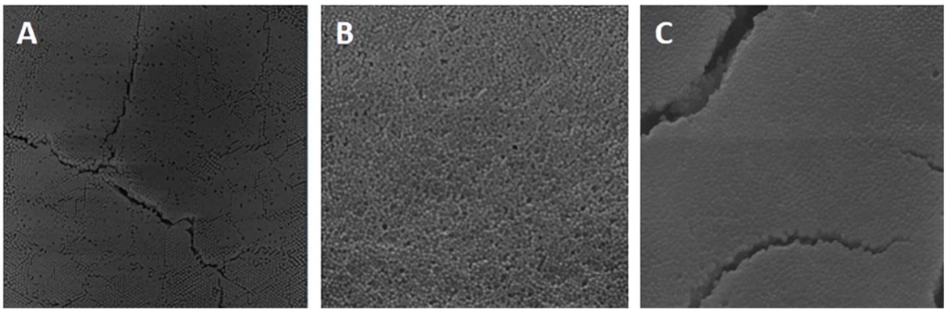


**Figure S5.** SEM images of polystyrene with different acrylic acid content: A) 0.2 wt% of AA, B) 0.4 wt% of AA, C) 0.6 wt% of AA.

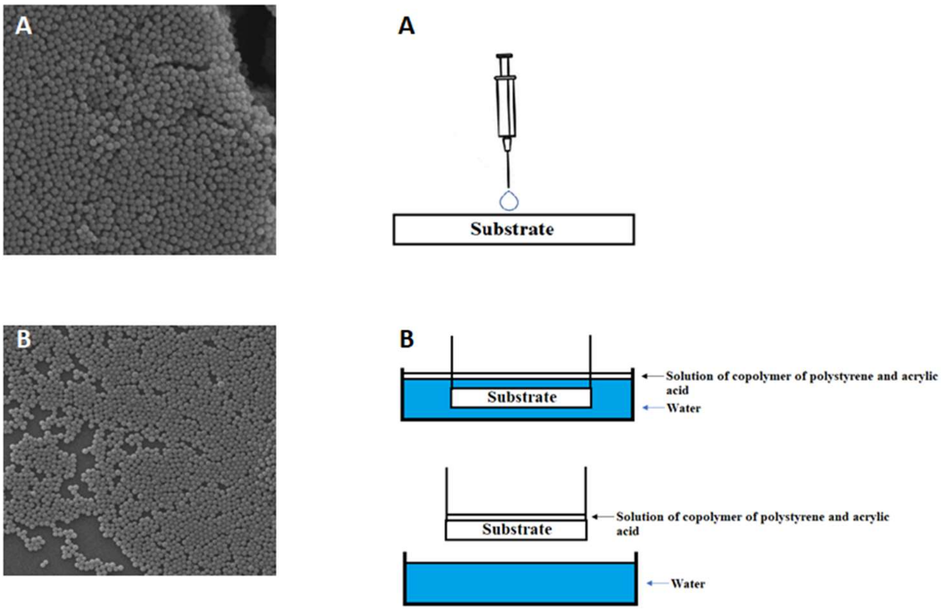


**Figure S6.** SEM images and scheme of deposition of polystyrene solution with 0.2 wt% acrylic acid content. (A) photonic crystals were obtained by adding polystyrene particles dropwise to the substrate; (B) photonic crystals were obtained by obtaining by removing the polystyrene film from water surface.

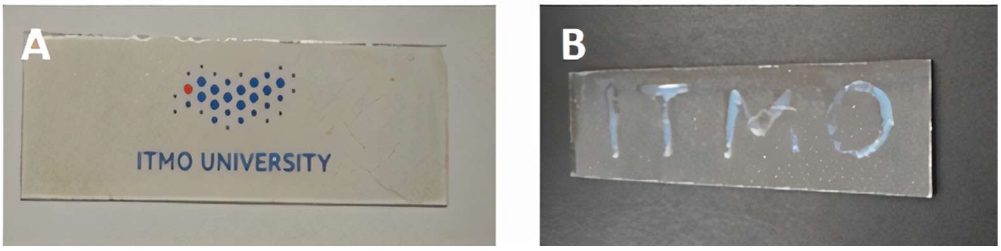


**Figure S7.** Photographs of a hydrogel before (A) and after (B) humidity changes. ITMO inscription was made with a wet brush.

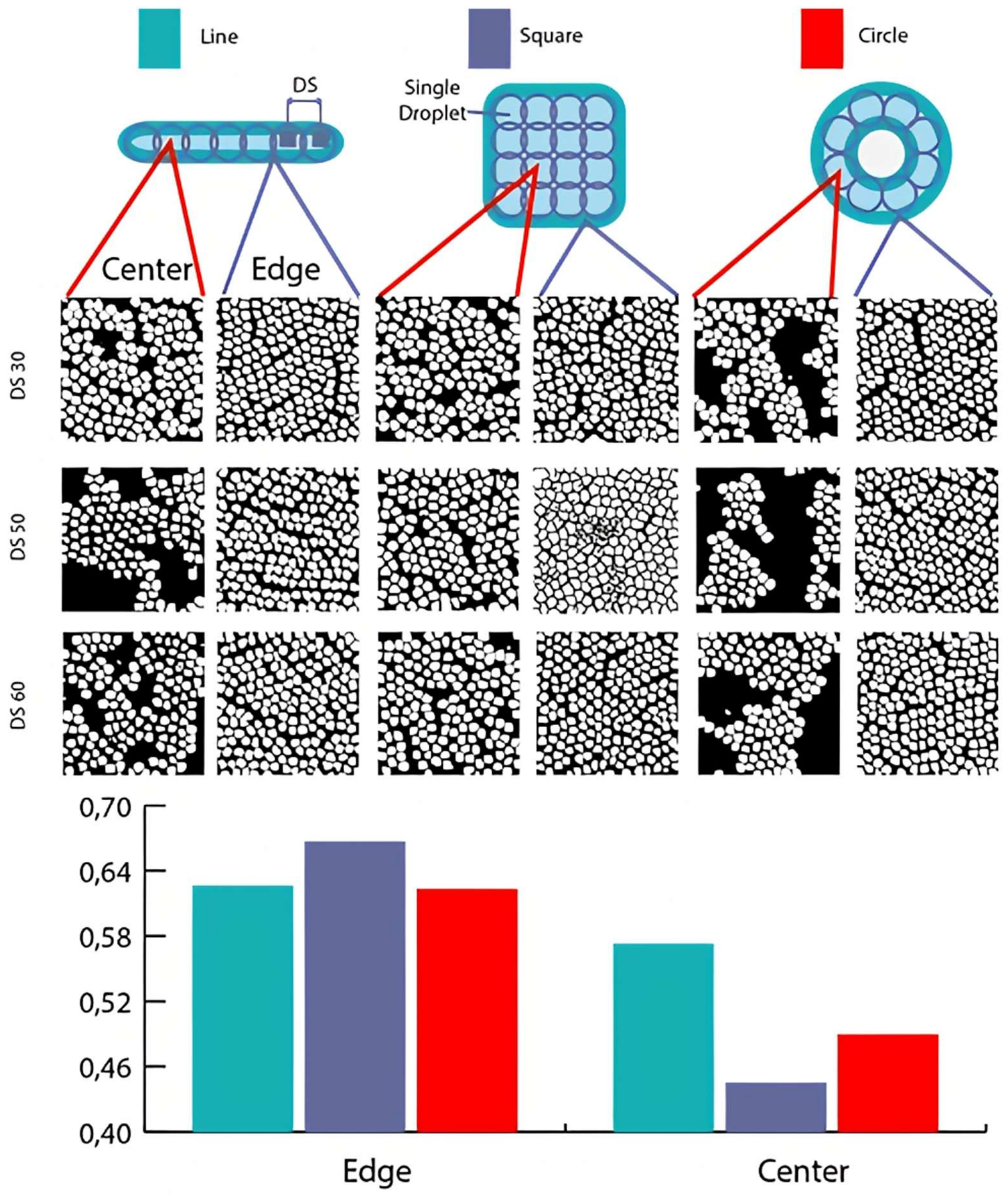


**Figure S8.** Image describing the dependence of the self-organization of polystyrene spheres on the shape of the printed pattern and the location of the spheres (center/ edge of the pattern).

**Table S1.** Different ratios of chitosan and glutaraldehyde for optimal hydrogel formation (**+** means that there was a swelling of the hydrogel after a change in humidity; after drying, the hydrogel assumed its original configuration; **-** means that there was no swelling either when the humidity changed or when completely submerged in water. This process was repeated several times). The optimal time for crosslinking the hydrogel is 10 seconds, then rinsing with water.

| Ratio of chitosan and glutaraldehyde | Chitosan purified NaOH | Chitosan washed with NaOH |
|---|---|---|
| 1 : 1 | - | - |
| 2 : 1 | - | - |
| 3 : 1 | - | - |
| 4 : 1 | + | - |
| 5 : 1 | + | + |
| 6 : 1 | + | + |
| 7 : 1 | + | + |

**Table S2.** Influence of hydrogel composition on sensitivity to humidity changes. **+** means that when humidity changes there is a color response and a change in the structure of the hydrogel, **-** means that when the humidity changes there is no change in the color of the hydrogel and the structure remains unchanged. This process was repeated several times.

| Ratio of PS to CS | Sensitivity of hydrogel to changes in humidity |
|---|---|
| 1 : 0.5 | - |
| 0.5 : 1 | + |
| 1 : 1 | + |
| 1 : 2 | + |
| 1 : 3 | + |
| 1 : 4 | +- |
| 1 : 5 | -+ |
| 1 : 6 | - |
